# Polymorphic low-grade neuroepithelial tumors of the young: disease characteristics and treatment decisions from the epilepsy surgery perspective

**DOI:** 10.3389/fneur.2024.1454056

**Published:** 2024-11-20

**Authors:** Guilu He, Hongping Tan, Shaochun Li, Limin Zhang, Qiao Li, Hainan Li, Yanwu Guo, Qiang Guo

**Affiliations:** ^1^Department of Neurosurgery, Zhujiang Hospital, Southern Medical University, Guangzhou, China; ^2^Department of Neurosurgery, Guangdong Sanjiu Brain Hospital, Guangzhou, China; ^3^Department of Pathology, Guangdong Sanjiu Brain Hospital, Guangzhou, China

**Keywords:** PLNTY, stereoelectroencephalography, epilepsy, neuroepithelial tumors, treatment

## Abstract

**Background:**

The Polymorphic Low-Grade Neuroepithelial Tumor of the Young (PLNTY) is a rare, epilepsy-associated brain tumor that has been increasingly recognized but is not well understood due to the scarcity of clinical reports. Our study reviews the clinical characteristics and treatment outcomes of 14 patients with PLNTY to enhance the understanding of this condition from an epilepsy surgery perspective.

**Methods:**

We performed a retrospective analysis of 14 PLNTY cases at our hospital. A literature review on prior studies was also conducted.

**Results:**

Our study included 8 males and 6 females, all presenting with epilepsy. Despite anti-seizure medication, 92.3% of patients continued to have seizures, with 58.3% diagnosed as having drug-resistant epilepsy. Neuroimaging revealed that 64.3% of the lesions were in the temporal lobe, with 75.0% showing calcification on CT, 71.4% exhibiting mixed signals on T2-weighted images, and 92.7% showing tumor enhancement. The transmantle sign was noted in 57.1% of T2 FLAIR sequences. EEGs indicated abnormal activity in 69.2% of patients, with 30.7% showing bilateral discharges. SEEG in two patients confirmed the tumor’s epileptogenicity. A 78.6% total resection rate was achieved, with a 90.0% postoperative seizure-free rate and an 85.7% excellent Engel grade 1 rate. No instances co-occurring with focal cortical dysplasia (FCD) were observed.

**Conclusion:**

PLNTY is characterized by unique neuroimaging features and a strong association with epilepsy. SEEG is pivotal for cases with unclear lateralization, aiding in identifying the link between the tumor and seizures. Following established epilepsy surgery protocols for brain tumor management, early intervention and extended resection can improve the rate of postoperative seizure freedom.

## 1 Introduction

PLNTY is a novel entity recently incorporated into the 2021 World Health Organization classification of central nervous system (CNS) tumors (1). It is distinguished by a significant association with epileptic seizures in adolescent individuals. Pathologically, PLNTY exhibits a diffuse growth pattern, scattered calcifications, and frequent oligodendroglioma-like components. Furthermore, immunohistochemistry reveals CD34 positivity and abnormal gene activation of the MAPK pathway. However, recent analyses suggest a considerable variability in its radiological and clinical presentation (1, 2). Given the limited number of reported clinical cases, a comprehensive understanding of the electro-clinical characteristics and treatment outcomes of this tumor is imperative. This study provides a detailed analysis of the clinical profiles and treatment courses of 14 pathologically confirmed PLNTY cases from our institution. We elucidate the unique features and therapeutic responses, supported by a relevant literature review.

## 2 Materials and methods

The medical records of all patients who underwent surgical treatment at our hospital from January 2021 to January 2024 were reviewed. In accordance with the Declaration of Helsinki, the Ethics Committee of Guangdong Sanjiu Brain Hospital does not require informed consent for the use of anonymous clinical data for retrospective analysis.

Data collection included demographic details, clinical presentations, diagnostic findings, extent of surgical resection, and therapeutic efficacy. The follow-up period begins after the individual’s surgery date, with a duration ranging from 3 months to 3 years to evaluate the outcomes of epilepsy treatment. The dataset was meticulously analyzed and interpreted by experienced epilepsy specialists. Due to the limited patient cohort, advanced statistical software was not necessary for the analysis.

A personalized surgical strategy was developed for each patient. The correlation between tumors and epileptic seizures was initially assessed using video-EEG monitoring. In cases where the localization of epilepsy was inconclusive or precise tumor resection was necessary, combined stereoelectroencephalography (SEEG) may be considered. Our institution utilizes a stereotactic planning system and MRI imaging to create a three-dimensional cerebral model, aiding in the precise delineation of the tumor and its adjacent anatomical structures. Intraoperatively, neuronavigation and electrocorticography (ECoG) are employed to guide the surgical resection. Postoperative MRI scans are performed to confirm the completeness of the resection.

Postoperative tissue samples were fixed in 10% neutral buffered formalin for subsequent immunohistochemistry (IHC) and molecular diagnostics. The IHC protocol targeted a panel of markers, including glial fibrillary acidic protein (GFAP), Oligodendrocyte transcription factor 2 (Olig2), B-type Raf kinase (BRAF), Cluster of Differentiation 34 (CD34), and Isocitrate Dehydrogenase 1 (IDH1). Samples with positive BRAF immunoreactivity underwent polymerase chain reaction (PCR) analysis to detect the BRAFV600E mutation.

## 3 Results

In this study, a total of 8 males (57.1%) and 6 females (42.9%) were included. The median age of onset was 19.3 years, ranging from 2.7 to 41 years. Three patients experienced disease onset after the age of 30, all of whom presented with temporal lobe lesions. Seizure was the initial symptom in all patients, with two cases also reporting auras. The duration of the disease ranged from 2 months to 18 years, with an average of 59.3 months. Prior to surgery, all patients had received varying degrees of anti-seizure drug therapy. Importantly, 92.3% (12 out of 13) of the patients had poorly controlled seizures, and among them, 58.3% (7 out of 12) met the criteria for refractory epilepsy. Detailed information regarding patient demographics and medication history can be found in Table 1.

**Table 1:** Basic data and clinical information of patients with PLNTY.

case	Sex	onset (years)	First neurological event/Duration (months)	Frequency	ASM tried	Response to ASM
1	F	6.8	Seizures/2	Daily	NA	No
2	F	15.3	Seizures/32	Monthly	VPA,CLN	No
3	M	20.6	Seizures/17	Weekly	VPA	No
4	M	22.3	Seizures/33	Daily	VPA,LEV,OXC	No
5	M	6.0	Seizures/36	Daily	OXC	No
6	F	25.0	Seizures/24	Monthly	LTG	No
7	M	2.7	Seizures/4	Weekly	OXC	Yes
8	M	37.2	Seizures/10	NA	NA	NA
9	F	18.0	Seizures/168	Monthly	VPA,CBZ	No
10	M	17.0	Seizures/216	Monthly	VPA,CBZ	No
11	F	30.0	Seizures/120	Daily	VPA,LTG	No
12	M	41.0	Seizures/60	Daily	VPA,OXC	No
13	F	28.0	Seizures/60	Monthly	OXC	No
14	M	10.0	Seizures/48	Daily	OXC, LTG, LEV, Lacosamide	No

NA, not available; VPA, Valproic Acid; CLN,Clonazepam; LEV, Levetiracetam; OXC, Oxcarbazepine; LTG, Lamotrigine; CBZ, Carbamazepine.

### 3.1 Imaging features

Temporal lobe lesions were found in 46.3% (9/14) of cases, with 3 cases involving the medial temporal lobe and 6 cases involving the lateral temporal lobe. Frontal lobe lesions were present in 14% (2/14) of cases, occipital lobe lesions in 14% (2/14), and parietal lobe lesions in 7% (1/14). The average tumor diameter was approximately 21.6 ± 8.8 mm (mean ± SD), with the largest tumor measuring 38 mm in diameter. On MRI, 71.4% (10/14) of tumors showed a mixed cystic-solid composition. Enhancement was observed on T1-weighted post-contrast sequences in 92.7% (11/12) of patients. Calcifications were detected on CT scans in 75.0% (9/12) of cases. Detailed information can be found in Table 2.

**Table 2:** Test data and related treatment results of PLNTY patients.

Case	Tumor location	EEG finding	T2Fsignals	Cystic component	Calculation	Contrast enhancement	transmantle singal	CD34	BRAF mutation	Treatment	Seizure outcome
1	L Parietal	Left posterior cortical	mixed	Yes	Yes	Yes	Yes	+	-	GTR	Seizure free
2	R Lateral temporal	Right temporal region	mixed	Yes	Yes	Yes	Yes	+	+	ER	Seizure free
3	L Occipital	Bilateral hemispheric discharge	mixed	No	Yes	NA	No	+	+	ER	Seizure free
4	R Frontal	Bilateral hemispheric discharge	mixed	No	Yes	Yes	No	+	-	GTR	NA
5	R Lateral temporal	Right frontal-temporal region	mixed	Yes	Yes	Yes	No	+	+	ER	Seizure free
6	R Lateral temporal	Right temporal region	iso	No	No	Yes	Yes	+	+	ER	NA
7	R Occipital	Right central-parietal–temporal region	mixed	Yes	Yes	Yes	Yes	+	-	PTR	Seizure free
8	L Lateral temporal	Bilateral hemispheric discharge	mixed	Yes	No	Yes	No	+	+	GTR	Seizure free
9	L Lateral temporal	Left temporal region	mixed	Yes	Yes	Yes	Yes	+	+	PTR	Uncontrolled
10	R Medial temporal	Bilateral hemispheric discharge	iso	Yes	No	none	Yes	+	+	ER	Seizure free
11	L Medial temporal	NA	hyper	Yes	NA	Yes	No	+	-	PTR	Seizure free
12	R Medial temporal	Right temporal region	hyper	No	NA	NA	Yes	+	-	GTR	NA
13	L Lateral temporal	Left temporal region	mixed	Yes	Yes	Yes	No	+	+	ER	NA
14	R Frontal	Right posterior cortical	hyper	Yes	Yes	Yes	Yes	+	-	ER	Seizure free

NA, not available; ER, enlarged resection; GTR, gross total resection; PTR, partial resection.

### 3.2 Electroencephalogram findings

**Figure 1:**
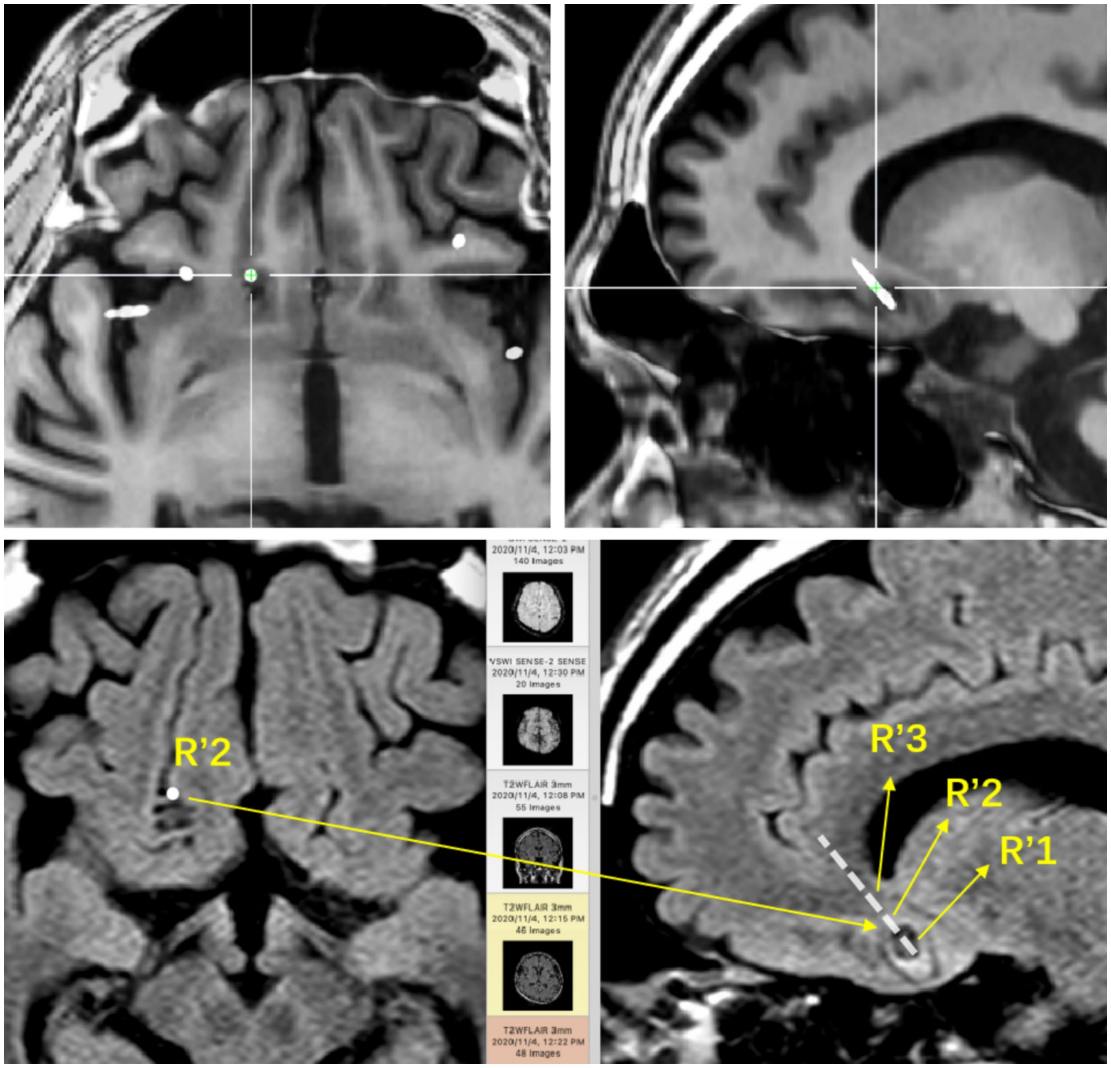
The base of the right frontal lobe. The positional relationship between the electrode contacts and the lesion entity: R’1 is located in the calcification of the lesion; R’2 is located in the parenchyma of the lesion; R’3 is located around the lesion.

**Figure 2:**
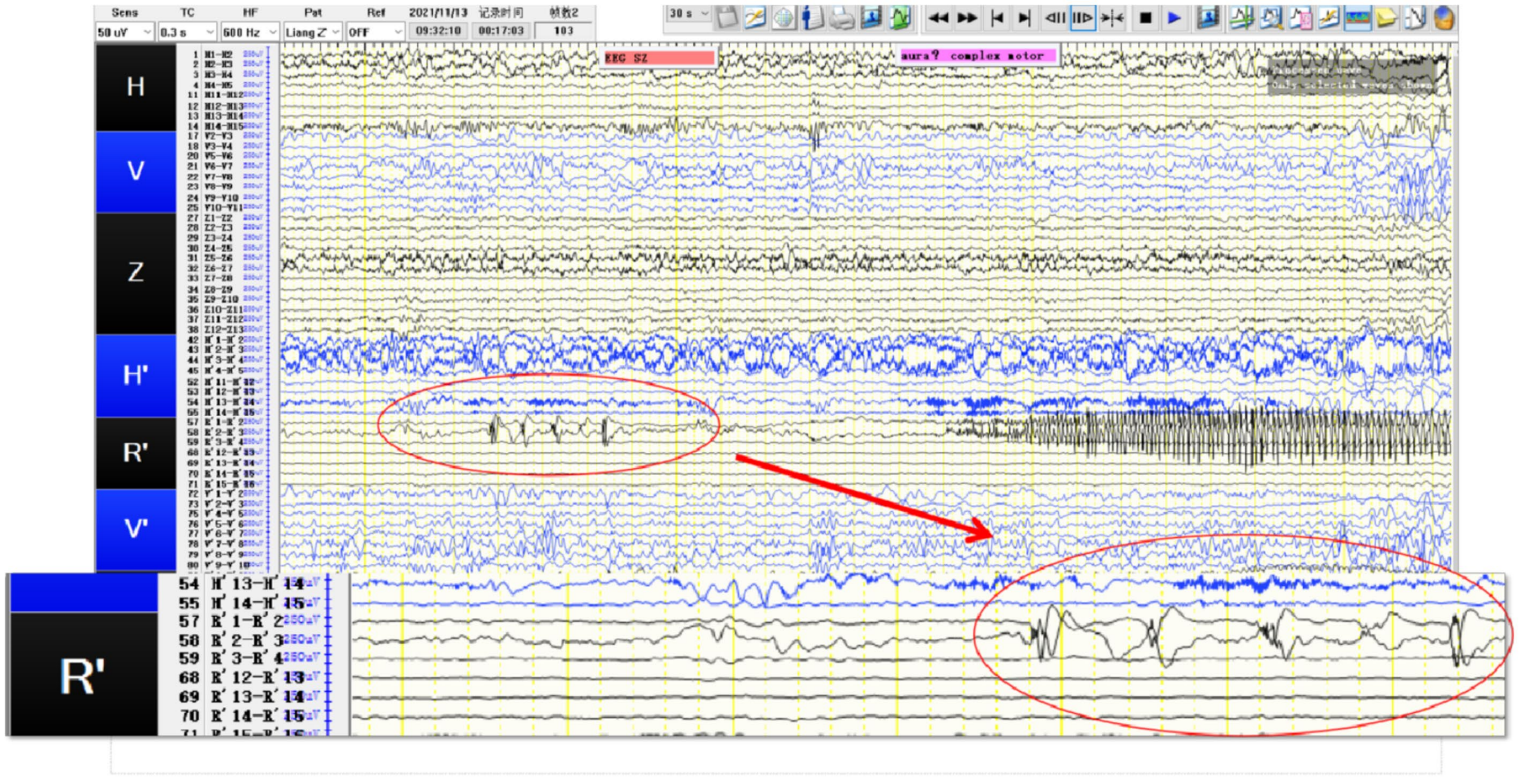
SEEG recording results: R’1 and R’2 are the seizure onset areas, while R’3 has not changed significantly. The tumor in this patient showed obvious intrinsic epileptogenicity, and the epileptogenic zone was only located within the tumor, while the surrounding tissues of the tumor showed no signs of seizure involvement.

**Figure 3:**
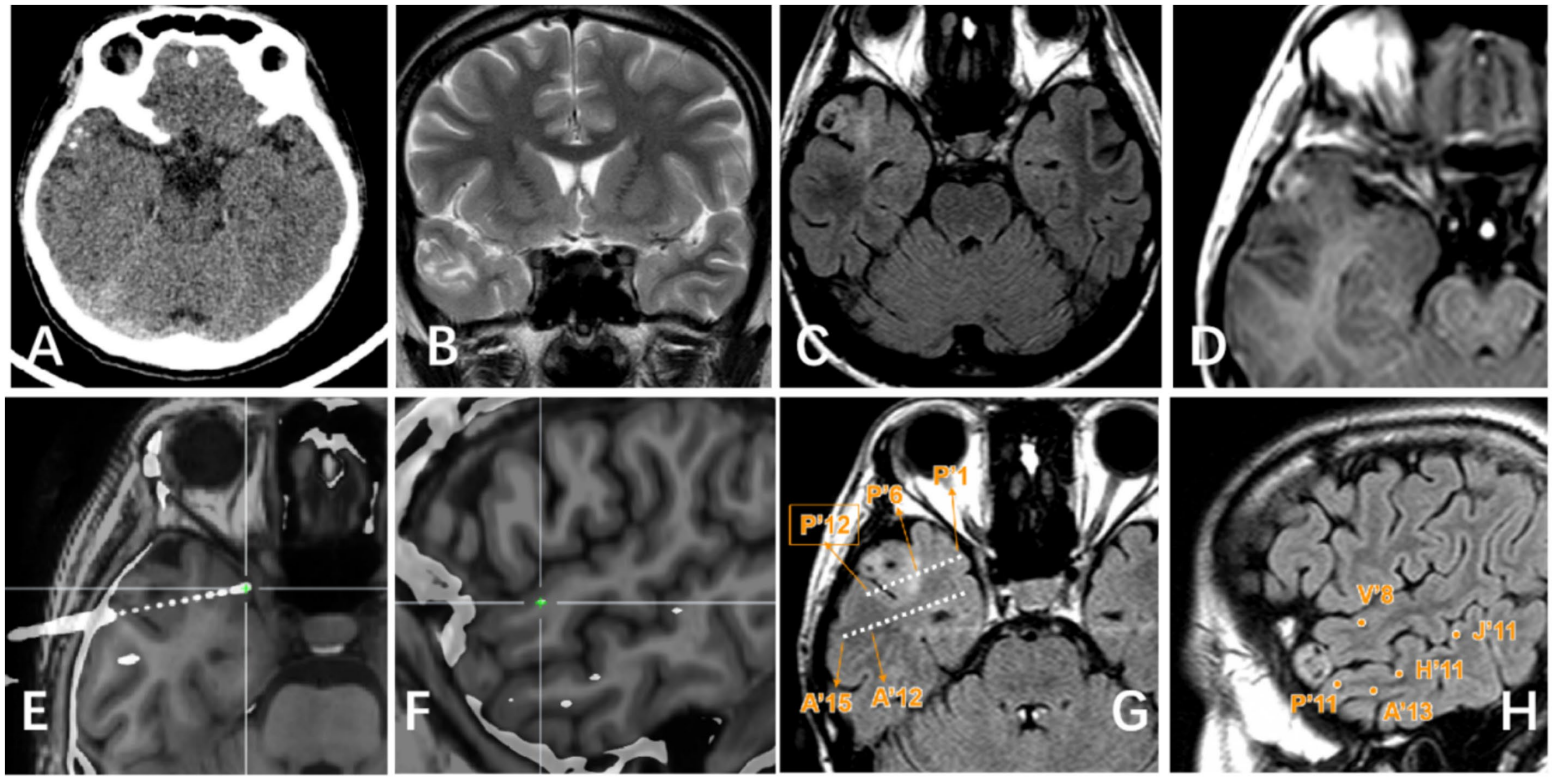
Right lateral temporal lobe lesion. (A) Scattered calcification in CT image; (B) Mixed signal in the lateral temporal lobe in T2WI image; (C) Transmantle sign changes can be seen in T2Flair; (D) Mild enhancement of tumor can be seen in T1 enhanced image; (E–H) Display the positional relationship between the electrode contact point and the tumor entity: P’6–12 is located in the tumor parenchyma and shows high signal on T2 Flair. Electrode contacts such as P’1–5/A’/H′/V′/J’ are located around the tumor and T2 Flair shows relatively normal signals.

**Figure 4:**
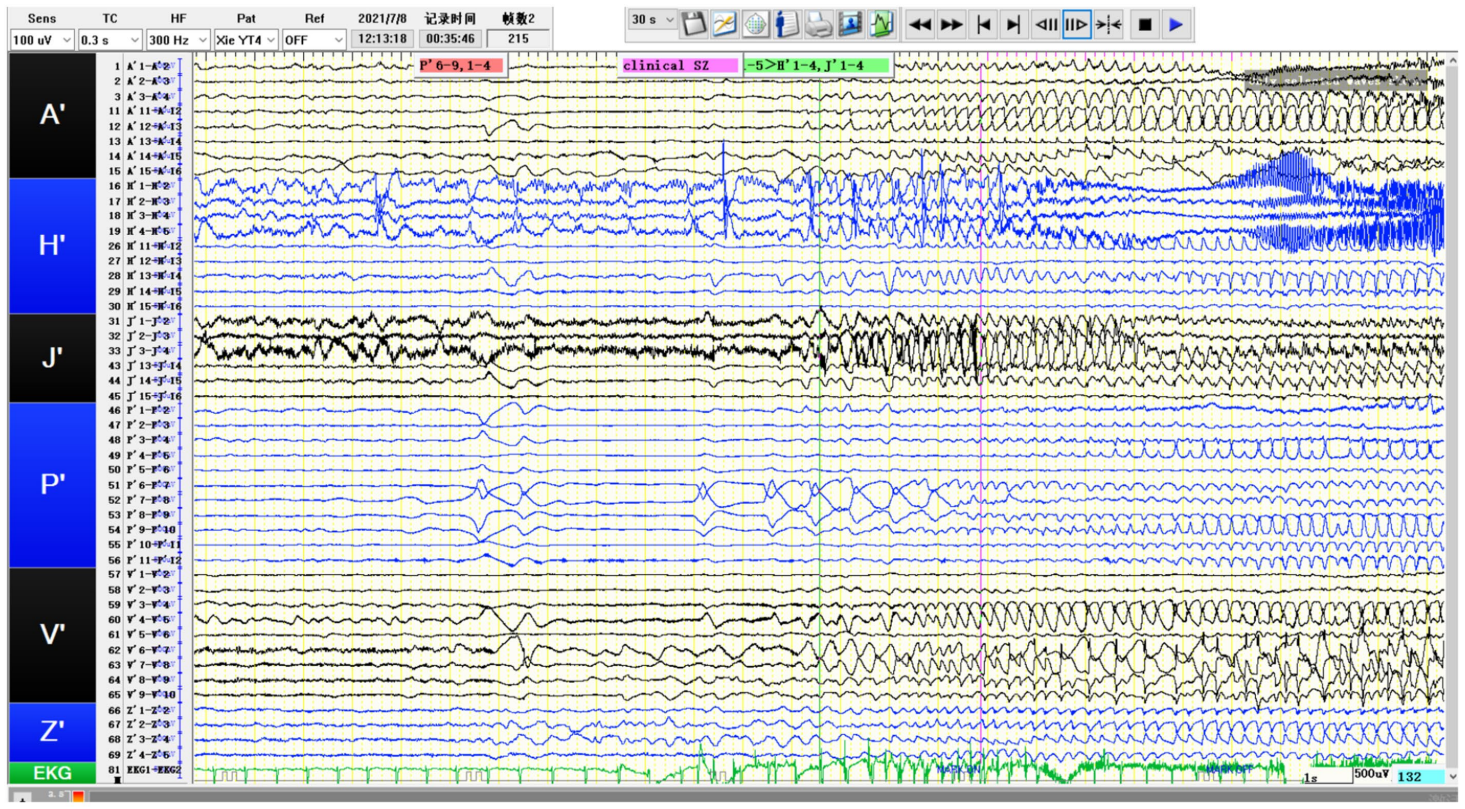
SEEG recording results: P’6–12 is the onset area of the seizure, while P’1-5/A’12-15/H’8-12/V’5-8/J’6–14 are rapidly affected in the early stage of the attack. The patient’s tumor showed obvious intrinsic epileptogenicity, and epileptic discharges rapidly spread to the surrounding tissues of the tumor at an early stage, especially the lateral neocortex and the medial hippocampus (H′1-3/J’1–3) not affected (still showing intermittent discharges).

The preoperative 24-hour EEG data were analyzed for 13 patients, revealing a 69.2% (9/13) concordance between EEG abnormalities and tumor locations. The remaining 4 patients showed bilateral abnormal discharges. Two patients underwent SEEG implantation. In Case 2, a pediatric patient with temporal lobe lesions opted for this invasive procedure to precisely define the resection margins at the parents’ request. Ictal SEEG recordings indicated significant intrinsic epileptogenicity within the tumor, with epileptic discharges rapidly spreading to the surrounding tissues, particularly affecting the lateral neocortex (Figures 1, 2). In Case 4, SEEG implantation was performed due to inconclusive lateralizing information from clinical symptoms, EEG, and PET scans (Figures 3, 4). During the ictal SEEG period, the tumor exhibited pronounced intrinsic epileptogenicity, with the epileptogenic zone strictly confined to the tumor’s interior. There were no signs of seizure involvement outside the tumor, including in the surrounding peripheral tissues.

### 3.3 Pathological results

In this cohort, all tumors exhibited positive expression for CD34 and GFAP (14/14, 100%), and were negative for IDH1. Regarding Olig-2 immunohistochemistry, one case initially considered weakly positive was ultimately classified as negative, resulting in a final positive expression rate of 92.9% (13/14) for Olig-2. In the BRAF assay, 57.2% of cases (8/14) showed positive expression. Among these, one case could not undergo further PCR analysis due to financial constraints. The remaining cases demonstrated a 100% (7/7) BRAFv600E mutation rate. No instances of these tumors co-occurring with focal cortical dysplasia (FCD) were observed In our cases. Detailed results can be found in Table 2.

### 3.4 Treatment outcome

Following postoperative MRI, complete tumor resection was successfully achieved in 78.6% of the cases. Three patients underwent partial resection due to the proximity of their lesions to eloquent brain regions, such as the lingual gyrus and corona radiata, necessitating a conservative surgical approach. During postoperative follow-up, “transmantle-like” signals were still evident on MRI in two patients, with one patient experiencing ongoing epileptic seizures. After surgery, complete seizure resolution was observed in 90.0% (9/10) of cases, and 85.7% (6/7) of patients attained Engel Class I status. Detailed outcomes are summarized in Table 2.

## 4 Discussion

PLNTY, a neuroepithelial tumor associated with epilepsy, was classified and categorized in the 2021 WHO classification of central nervous system neoplasms (1, 2). In this review, the age of onset ranged from 2.7 to 41 years, with an average of 20 years. The longest documented disease duration was 18 years, consistent with historical data (3). Unlike previous reports where 82.2% of patients presented with epileptic seizures as the initial symptom (3), all patients in our cohort presented with seizures. Table 1 provides detailed information on anti-seizure medication regimens and therapeutic responses. The high rate of drug resistance observed in our series highlights the importance of early surgical intervention in managing these tumors.

In our case series, PLNTY exhibited three notable imaging characteristics: cystic changes, calcification, and contrast enhancement, corresponding to proportions of 71.4% (10/14), 75.0% (9/12), and 92.7% (11/12), respectively. These tumors were most commonly located in the temporal lobe, particularly the lateral temporal region, and appeared as solid or mixed solid-cystic lesions. Cystic components varied in size, ranging from subtle punctate signal changes to more substantial eccentric cystic expansions. The cystic areas were typically well-demarcated compared to the solid portions, and the tumors rarely caused mass effect or peritumoral edema. After contrast administration, the tumors often showed mild, indistinct patchy or mottled enhancement patterns. This observed rate of enhancement is significantly higher than the previously reported 33% (4), which may be attributed to subjective differences in imaging assessments among various researchers. Calcifications were observed as central dense opacities or sparse lamellar calcifications at the tumor’s edge. Besides, it appears as hypometabolism during the interictal phase on PET-CT.

There is limited reporting on EEG characteristics related to PLNTY. Our study shows that the discharge patterns exhibited by PLNTY are similar to those of most brain tumor-related epilepsy. Scalp EEG indicates that the tumor-related discharge sites are consistent with the lobe of the brain where the tumor is located, but there is a certain proportion of cases with discharges involving both cerebral hemispheres. Additionally, we observed significant intrinsic epileptogenicity of the tumor in 2 patients who underwent SEEG. However, the presence of early rapid spread of electroencephalographic activity was observed to be different. This observation differs from previous descriptions, which noted epileptic discharge areas around PLNTY deviating from the tumor’s center (5). It suggests that for PLNTY with strong epileptogenicity, expanding the resection scope to relatively safe areas may be advantageous for epilepsy control.

Similar to the management of other epilepsy-related brain tumors, surgical resection is the primary therapeutic approach for PLNTY, involving the removal of both the tumor and associated epileptogenic foci. A comprehensive preoperative evaluation, including clinical symptoms, EEG data, and understanding of epileptogenic network pathways, is crucial for establishing the link between the tumor and epilepsy and defining surgical margins. In cases where there is a discrepancy between the epileptogenic zone identified by non-invasive methods and the tumor boundaries delineated by MRI, invasive intracranial electroencephalography with depth electrodes may be employed to determine their correlation (3, 5).

In our series of cases, patients who underwent complete or extended resections were seizure-free postoperatively. It is noteworthy that PLNTY often presents with “Transmantle-like” peritumoral signal changes on MRI, and the persistence of these signal abnormalities post-resection is significantly correlated with unfavorable epilepsy control outcomes (3). Achieving total tumor resection is paramount for optimizing the prognosis of patients with epilepsy. “Transmantle-like” changes were observed in 57.1% (8/14) of our cases. Due to the proximity of these changes to critical functional areas, only partial resections were performed in two patients, and one of them, with a medical history of 168 months, experienced early postoperative seizures.

A literature review by Armocida et al., which examined 51 cases, identified a significant association between tumor contrast enhancement on MRI and adverse postoperative epilepsy outcomes (2). In our series, 92.7% (11/12) of patients exhibited contrast enhancement on T1-weighted imaging, with a 90.0% (9/10) rate of complete seizure resolution postoperatively, and 85.7% (6/7) achieving Engel Class I status. Our follow-up data suggest that surgical tumor resection has a definitive impact on improving drug-resistant epilepsy in patients. However, the correlation between MRI contrast enhancement and postoperative epilepsy control outcomes necessitates further validation with a larger sample size.

PLNTY is characterized by its unique pathological features, with CD34 positive expression and BRAF V600E mutation being its most distinctive alterations (2). Research indicates that CD34-positive tumor cells can trigger an inflammatory response, leading to inflammation and damage to surrounding brain tissue, thereby increasing the risk of epileptic seizures, which clinically manifests as a significant association with a longer history of epilepsy (6). In this series, no instances co-occurring with focal cortical dysplasia (FCD) were observed. Nearly all patients appearance of structural disorder in the center of the tumor and the adjacent cortex, and there is also a dendritic positive pattern of immunohistochemical CD34. We believe this represents the tumor’s involvement of the surrounding cortex rather than type FCDIIIb. Our findings may imply the tumor’s invasion of the surrounding brain tissue, explaining the necessity of early intervention and extensive tumor resection from a pathological standpoint.

The BRAF mutation is associated with various types of epilepsy (7). The expression of mutated BRAF protein may impact neural networks, causing abnormal discharges in different neuronal populations in different locations, potentially resulting in various epileptic patterns. BRAF V600E is the most prevalent type of activating BRAF mutation and is linked to the development of epileptic activity mediated by alterations in ion transport and synaptic activity regulation. There is a perspective that tumors with positive BRAF V600E expression may represent a distinct subset of PLNTY tumors, exhibiting a more indolent course, later age of onset, and a delay in clinical symptom presentation (2). In our case series, 57.2% of patients tested positive for BRAF V600E. The average age of onset for BRAF V600E-positive patients is 20.6 years, with 87.5% (7 out of 8) of these tumors located in the temporal lobe. This proportion closely aligns with Baumgartner’s reported 87.1% of BRAF V600E-positive tumors involving the temporal lobe (2). This observation may suggest a specific association between the BRAF V600E mutation and the occurrence of temporal lobe tumors, warranting further attention in future mechanistic research.

## 5 Limitations

This article reports on a retrospective study. The lack of detailed records of intraoperative monitoring results limits our ability to extensively discuss this aspect. Additionally, the small number of cases, single-center sampling, and short follow-up periods for some patients may restrict our capacity to fully assess the treatment effects, which in turn limits the scope and depth of our study. Future research should aim to expand the sample size, prolong the follow-up period, and focus on intraoperative monitoring to better understand the neuroelectric relationships between tumors and surrounding tissues.

## 6 Conclusion

PLNTY is known for its high epileptogenicity and distinctive imaging features. SEEG implantation may be suitable for elucidating the relationship between non-lateralized tumors and epileptic seizures. Early intervention and extended resection appear to increase the rate of postoperative seizure freedom.

## Data Availability

The original contributions presented in the study are included in the article/supplementary material, further inquiries can be directed to the corresponding author.
